# Hodgkin lymphoma treatment with ABVD in the US and the EU: neutropenia occurrence and impaired chemotherapy delivery

**DOI:** 10.1186/1756-8722-3-27

**Published:** 2010-08-19

**Authors:** Matthias Schwenkglenks, Ruth Pettengell, Thomas D Szucs, Eva Culakova, Gary H Lyman

**Affiliations:** 1 Institute of Pharmaceutical Medicine, University of Basel, Basel, Switzerland; 2 St George's University of London, London, UK; 3 Duke University, Durham, North Carolina, USA

## Abstract

**Background:**

In newly diagnosed patients with Hodgkin lymphoma (HL) the effect of doxorubicin, bleomycin, vinblastine and dacarbazine (ABVD)-related neutropenia on chemotherapy delivery is poorly documented. The aim of this analysis was to assess the impact of chemotherapy-induced neutropenia (CIN) on ABVD chemotherapy delivery in HL patients.

**Study design:**

Data from two similarly designed, prospective, observational studies conducted in the US and the EU were analysed. One hundred and fifteen HL patients who started a new course of ABVD during 2002-2005 were included. The primary objective was to document the effect of neutropenic complications on delivery of ABVD chemotherapy in HL patients. Secondary objectives were to investigate the incidence of CIN and febrile neutropenia (FN) and to compare US and EU practice with ABVD therapy in HL. Pooled data were analysed to explore univariate associations with neutropenic events.

**Results:**

Chemotherapy delivery was suboptimal (with a relative dose intensity ≤ 85%) in 18-22% of patients. The incidence of grade 4 CIN in cycles 1-4 was lower in US patients (US 24% vs. EU 32%). Patients in both the US and the EU experienced similar rates of FN across cycles 1-4 (US 12% vs. EU 11%). Use of primary colony-stimulating factor (CSF) prophylaxis and of any CSF was more common in the US than the EU (37% vs. 4% and 78% vs. 38%, respectively). The relative risk (RR) of dose delays was 1.54 (95% confidence interval [CI] 1.08-2.23, *p *= 0.036) for patients with vs. without grade 4 CIN and the RR of grade 4 CIN was 0.35 (95% CI 0.12-1.06, *p *= 0.046) for patients with vs. without primary CSF prophylaxis.

**Conclusions:**

In this population of HL patients, CIN was frequent and FN occurrence clinically relevant. Chemotherapy delivery was suboptimal. CSF prophylaxis appeared to reduce CIN rates.

## Introduction

Combination therapy with doxorubicin, bleomycin, vinblastine and dacarbazine (ABVD) is the standard chemotherapy regimen for patients with Hodgkin lymphoma (HL) [1-3]. Myelosuppression, in particular neutropenia, is common during ABVD treatment [2]. Chemotherapy-induced neutropenia (CIN) can lead to febrile neutropenia (FN), which is associated with considerable morbidity, mortality and costs [4]. Standard care for the majority of FN patients requires hospitalisation and administration of intravenous antibiotics [5,6].

Neutropenic events often result in dose delays and dose reductions, leading to impaired chemotherapy delivery which has been associated with decreased survival in certain types of cancer [7-10], indicating that optimal intensity of chemotherapy treatment can improve patient outcomes [8]. Colony-stimulating factors (CSFs) have been shown to reduce the incidence and severity of neutropenic events across a broad range of malignancies and regimens and also to support the delivery of full chemotherapy dose intensity [5,11].

In patients with HL, the effect of ABVD-related neutropenia and neutropenic complications on chemotherapy delivery are poorly documented [2,12]. Two similarly designed, prospective, observational studies were conducted in the US [13] and Europe [14] to assess the incidence of neutropenia in patients undergoing chemotherapy. Here we present a subgroup analysis of HL patients from these studies. The primary objective was to assess the effects of neutropenic complications on the delivery of ABVD chemotherapy. Secondary objectives were to investigate the incidence of CIN and FN in patients with HL undergoing ABVD chemotherapy and to compare US and EU practice with ABVD therapy in HL.

## Methods

Two similarly designed, prospective, observational studies [13,14] enrolled patients with solid tumours or lymphoma initiating a new course of chemotherapy, with at least 4 cycles planned, during the period 2002-2005. In the US, a total of 4458 patients were recruited from 115 community practices. In the EU, a total of 749 patients were recruited from 66 clinical centres in Belgium, France, Germany, Spain and the UK.

Patients eligible for inclusion in this subgroup analysis were adults aged ≥ 18 years about to start a new course of ABVD (patients in whom doxorubicin was replaced with epirubicin were also allowed). In the US study, patients had a minimum life expectancy of at least 3 months. In the EU study, patients had to be HL stage IB-IV. Prior chemotherapy and concurrent radiation therapy were permitted. Key exclusion criteria were: use of antibody-based or cell-based immunotherapies, a history of stem-cell or bone-marrow transplantation and HIV infection. Additionally, the US study excluded patients diagnosed with myeloma or treated for active infection and did not allow participation in double-blind clinical trials. Patients in the EU were excluded if they had conditions causing neutropenia, malignant conditions with myeloid characteristics, or active infection within 72 hours prior to the start of chemotherapy. Concurrent participation in phase I/II clinical trials was not permitted. Ethical approval was obtained for all centres and all participants provided informed consent.

Data were merged and variable definitions reconciled to form a single, pooled dataset. Body surface area was calculated using the Mosteller formula [15]. Delivery of chemotherapy was assessed by considering the proportion of patients that received relative dose intensity (RDI) ≤ 85% of the planned or standard dose intensity and by documenting the occurrence of dose reductions > 10% and dose delays > 3 days. As delivery of vinblastine is unlikely to be affected by neutropenia, this agent was excluded from the calculation of RDI and dose reductions. In the US study, blood counts were drawn at the beginning of each cycle and at mid-cycle, for up to 4 cycles of treatment. In the EU study, a blood count at the expected (protocol defined) absolute neutrophil count (ANC) nadir was required in cycle 1. Centres were also required to record all blood counts taken during each patient's chemotherapy treatment. Grades 3 and 4 CIN were defined as an ANC < 1000/mm^3 ^and < 500/mm^3 ^[16], respectively, and FN as ANC < 1000/mm^3 ^in combination with site-reported fever above 38°C and/or infection. Primary CSF prophylaxis was defined as CSF use in the first cycle of chemotherapy before a documented grade 3-4 CIN occurred or denoted as primary prophylaxis by the site.

Due to the limited sample size, analyses were predominantly descriptive. Univariate associations between variables were explored in the pooled dataset. Associations of binary data were expressed as relative risks with accompanying 95% confidence intervals. Significance testing was based on Fisher's exact test (2-sided) due to small sample size, which explains some apparent inconsistencies between *p *values and confidence limits.

## Results

### Patient characteristics

A total of 115 HL patients (68 US patients, 47 EU patients) met the eligibility criteria and were included in the analysis. The age range was 19-83 years (median 36) in US patients and 18-74 years (median 34) in EU patients; 49% of US patients and 38% of EU patients were female. US patients had slightly higher body surface area and higher incidence of stage III/IV disease than EU patients and were more often pre-treated with radiotherapy (Table 1). Eastern Cooperative Oncology Group performance status was similar between US and EU patients and no patients had prior chemotherapy.

**Table 1:** Patient, disease and treatment characteristics

Characteristic		US (N = 68)	EU (N = 47)
Age in years, mean ± SD (range)		40.9 ± 16.2 (19-83)	37.9 ± 16.5 (18-74)
Female gender, N (%)		33 (48.5)	18 (38.3)
Race, N (%)	Caucasian/white	54 (79.4)	46 (97.9)
	Black	10 (14.7)	0 (0.0)
	Other	4 (5.9)	1 (2.1)
BSA at baseline in m^2^, mean ± SD (range)		1.94 ± 0.26 (1.42-2.53)	1.85 ± 0.21 (1.41-2.28)
ECOG status, N (%)	0	47 (69.1)	30 (63.8)
	1	20 (29.4)	14 (29.8)
	2	1 (1.5)	3 (6.4)
Disease stage ^1^, N (%)	I	8 (12.1) ^2^	5 (10.6)
	II	30 (45.5) ^2^	28 (59.6)
	III	23 (34.8) ^2^	8 (17.0)
	IV	5 (7.6) ^2^	6 (12.8)
Prior radiotherapy, N (%)		6 (8.8)	0 (0.0)
Baseline WBC in 10^3^/mm^3^, mean ± SD; median		9.4 ± 4.9; 7.8	9.5 ± 3.8; 8.4
Baseline ANC in 10^3^/mm^3^, mean ± SD; median		6.5 ± 3.3; 5.3 ^2^	7.2 ± 3.6; 6.6
Diabetes, N (%)		8 (11.8)	0 (0.0)
Cardiac comorbidity, N (%)		0 (0.0)	2 (4.3)
Planned dose intensity in mg/m^2^/week, mean ± SD; median			
Bleomycin		4.9 ± 0.9; 4.9	5.3 ± 1.2; 5.0
Doxorubicin		12.5 ± 1.9; 12.3	12.8 ± 2.7; 12.4^3^
Dacarbazine		184.0 ± 29.5; 183.6	198.3 ± 50.2; 186.7
Vinblastine		3.0 ± 0.5; 2.9	3.2 ± 0.8; 3.0
Planned cycle number, N (%)	≤ 3	0 (0.0)	2 (4.3)
	4-5	29 (42.6)	16 (34.0)
	6	38 (55.9)	26 (55.3)
	≥ 8	1 (1.5)	3 (6.4)
Planned cycle length in days, N (%)	14	7 (10.3)	5 (10.6)
	21	5 (7.4)	2 (4.3)
	28	56 (82.4)	40 (85.1)

*BSA *body surface area; *ECOG *Eastern Cooperative Oncology Group; *WBC *white blood cell count; *ANC *absolute neutrophil count.

^1 ^US: based on American Joint Committee Cancer staging; EU: based on Ann Arbor staging.

^*2 *^N = 66 due to missing values.

^*3*^N = 44 as 3 EU patients received epirubicin.

### Treatment characteristics

In most patients, 4-5 or 6 cycles of ABVD were planned. In the US and the EU, median planned dose intensities (expressed on the basis of actual body weight) met the ABVD standard of bleomycin, 5 units/m^2^/week; doxorubicin, 12.5 mg/m^2^/week; dacarbazine, 187.5 mg/m^2^/week; and vinblastine, 3 mg/m^2^/week. Actual planned dose intensities deviated in a number of patients and resulting means were marginally higher in the EU patients (Table 1). One US patient (1.5%) and three EU patients (6.4%) received epirubicin instead of doxorubicin (EBVD).

The percentage of patients receiving CSF overall and as primary prophylaxis was higher for US patients (any CSF use: US 78% vs. EU 38%; primary CSF prophylaxis: US 37% vs. EU 4%). Antibiotic use was similar between the two populations (any antibiotic use: US 41% vs. EU 49%; primary prophylaxis with antibiotics: US 13% vs. EU 17%).

### Chemotherapy delivery

**Figure 1:**
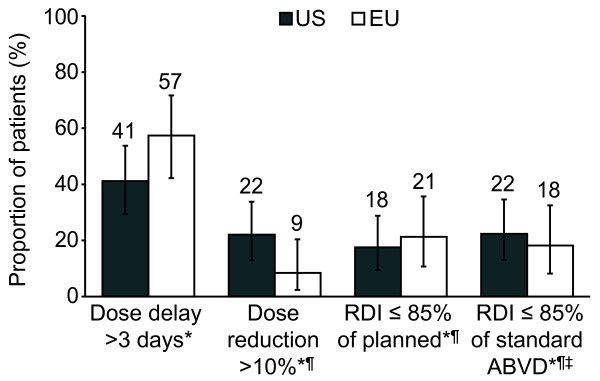
**Chemotherapy delivery in US and EU patients**. Incidence of dose delays > 3 days in any cycle, dose reductions > 10% in any drug in any cycle, and RDI ≤ 85% compared to either planned RDI or standard ABVD in US (N = 68) and EU (N = 47) patients during the first 4 cycles of chemotherapy. Error bars represent 95% CIs. *Assessment took into account administered cycles only; ^¶^Disregarding vinblastine; ^‡^EBVD patients excluded (US N = 67, EU N = 44); Standard ABVD: bleomycin 5 units/m^2^/week, doxorubicin 12.5 mg/m^2^/week, dacarbazine 187.5 mg/m^2^/week, vinblastine 3 mg/m^2^/week. *RDI *relative dose intensity; *ABVD *doxorubicin, bleomycin, vinblastine and dacarbazine; *CI *confidence interval; *EBVD *epirubicin, bleomycin, vinblastine and dacarbazine.

Dose delays > 3 days were more frequently observed in EU patients and dose reductions > 10% were more frequent in US patients (Figure 1). Chemotherapy delivery was suboptimal in 18-22% of patients (RDI ≤ 85% of ABVD standard). Comparison against the actual planned dose intensity for each individual patient led to a very similar result.

### Incidence of neutropenia and FN

**Figure 2:**
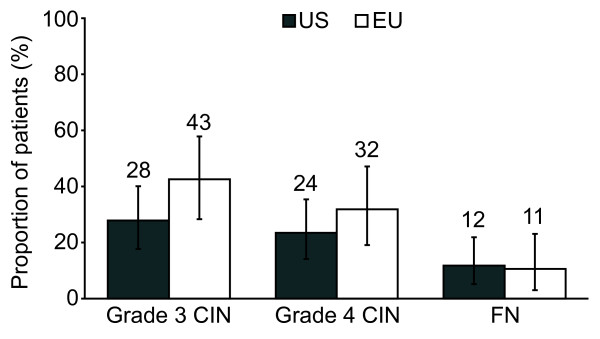
**Incidence of neutropenic events in US and EU patients**. Incidence of grade 3 and 4 CIN and FN in US (N = 68) and EU (N = 47) patients during the first 4 cycles of chemotherapy. Patients with grade 4 CIN were not counted as having grade 3 CIN. Error bars represent 95% CIs. *CIN *chemotherapy-induced neutropenia; *FN *febrile neutropenia; *CI *confidence interval.

Patients in both the US and the EU experienced similar rates of FN in the first cycle of chemotherapy (US 7% vs. EU 9% EU) and across cycles 1-4 (US 12% vs. EU 11%). The incidence of CIN in cycles 1-4 was lower in US patients (Figure 2). US patients had a mean ANC nadir of 2000 ± 2300/mm^3 ^in the first cycle compared to EU patients whose mean ANC nadir was 1300 ± 1000/mm^3 ^in the first cycle.

### Factors associated with chemotherapy delivery in the pooled dataset

The relative risk (RR) of dose delays > 3 days was 1.54 (95% confidence interval [CI] 1.08-2.23, *p *= 0.036) for patients with vs. without grade 4 CIN. There was no evidence of an association between the presence of grade 4 CIN in any cycle and dose reductions > 10% or RDI ≤ 85% of planned/standard. Similarly, there was no evidence of an association between grade 4 CIN in cycle 1 and dose delays, dose reductions or RDI ≤ 85% of planned/standard. CSF primary prophylaxis was not associated with dose delays > 3 days, dose reduction > 10% or RDI ≤ 85% of planned/standard.

### Association of CSF prophylaxis and neutropenic events in the pooled dataset

Patients receiving CSF primary prophylaxis were less likely to develop CIN than patients who did not receive CSF primary prophylaxis. The RR of grade 4 CIN in any cycle was 0.35 (95% CI 0.12-1.06, *p *= 0.046) for patients with vs. without primary CSF prophylaxis, and the RR of grade 3 or 4 CIN in any cycle was 0.42 (95% CI 0.23-0.76, *p *< 0.001). There was also a reduced risk of grade 3 or 4 CIN in cycle 1 for patients with vs. without CSF prophylaxis (RR 0.40, 95% CI 0.19-0.83, *p *= 0.004). There was no evidence of an association between CSF primary prophylaxis and incidence of FN in cycle 1 or cycles 1-4. In cycle 1, FN incidence was 7% in 27 patients with CSF primary prophylaxis and 8% in 88 patients with no CSF primary prophylaxis (RR 0.93, 95% CI 0.21-4.22, *p *= 1.000). In cycles 1-4, corresponding incidences were 15% and 10% (RR 1.45, 95% CI 0.48-4.33, *p *= 0.500). The other univariate associations considered were not statistically significant.

### Discussion

This study assessed the impact of CIN on ABVD chemotherapy delivery in HL patients in the EU and the US. Baseline characteristics were similar in both groups although US patients had a more advanced disease state. In both EU and US patients, CIN occurrence was substantial and the observed FN incidence of 11-12% was considerably higher than the 4% reported in current European Organisation for Research and Treatment of Cancer (EORTC) guidelines [5]. EORTC guidelines are based on a literature review of clinical trial data and under-reporting of febrile events has been noted to be common in randomised controlled trials [17]. This study is the first multi-centre investigation of neutropenic event incidence in general populations of HL patients treated with ABVD. Three retrospective single-centre studies have also addressed this topic [18-20]. Populations studied were similar to ours with respect to median age and grade 3/4 CIN risk per patient. However, grade 3/4 CIN risk per patient was not available from Evens et al. [18], and in the single-physician experience (with no CSF use) reported by Boleti and Mead, the proportion of stage III-IV patients was only 13% [19]. Overall FN incidence was 10%, 5-9% and 5% in the studies by Chand et al. (N = 81) [20], Evens et al. (N = 84) [18] and Boleti and Mead (N = 38) [19], respectively. These findings are not incompatible with our results, considering that retrospective data may be affected by incomplete recording. In addition, practice patterns can differ, and chance effects may play a role in small patient samples.

In both the US and EU populations, chemotherapy delivery was suboptimal with 18-22% of patients receiving RDI ≤ 85% compared to standard/planned. As the importance of ABVD dose intensity in determining remission and survival has not yet been defined [2], the clinical impact of this suboptimal ABVD delivery is not known. However, the data highlight that impaired chemotherapy delivery remains a problem in everyday clinical practice, although single centres may achieve very high average chemotherapy dose intensity [18]. Univariate analysis showed that grade 4 CIN increased the risk of dose delays > 3 days; however, the small patient numbers in each data set did not allow for efficient multivariate adjustment to assess the link between neutropenia and compromised chemotherapy delivery. Moreover, due to incomplete timing information, we could not clearly establish which dose delays and dose reductions occurred before or after neutropenic events, which may have diluted some associations. The influence of reduced or delayed chemotherapy delivery on neutropenic event occurrence remains to be assessed in HL patients receiving ABVD.

Use of primary CSF prophylaxis in ABVD patients was more common in the US than the EU, and in the univariate analysis performed, CSF prophylaxis was associated with a reduced risk of grade 4 CIN. However, the numbers of patients in each dataset were too small for efficient multivariate analysis of CIN risk. Despite greater CSF use and more dose reductions in the US population, similar FN rates were observed between patients in the EU and the US. This may be explained by a more advanced disease state in US patients, which has been identified as an adverse risk factor for increased incidence of FN [5].

In summary, CIN was frequent and FN occurrence clinically relevant in HL patients receiving ABVD chemotherapy. Dose delays and dose reductions were frequent and resulted in suboptimal delivery of chemotherapy in approximately one fifth of patients. Use of primary CSF prophylaxis was more common in the US than the EU and appeared to reduce CIN rates.

## Authors Information

MS, RP, and TDS: On behalf of the Impact of Neutropenia in Chemotherapy - European Study Group (INC-EU).

EC and GHL: On behalf of the Awareness of Neutropenia in Chemotherapy Study Group (ANC)

## Competing interests

RP has received honoraria from Amgen, Bayer and Roche and has been a paid expert for Amgen, Bayer and Roche.

MS has received honoraria and research funding from Amgen and has acted as a consultant for Amgen.

GHL has been a PI on a research grant from Amgen to the Duke University in support of the ANC Study Group and has received honoraria from Amgen.

EC and TDS have no competing interests.

## Authors' contributions

RP, MS and TDS were involved in the collection and interpretation of INC-EU prospective study data. EC and GHL were involved in the collection and interpretation of ANC prospective study data. MS performed the data analysis presented here. RP, MS, TDS, EC and GHL participated in drafting the manuscript. All authors read and approved the final manuscript.

## References

[B1] Duggan DB Petroni GR Johnson JL Glick JH Fisher RI Connors JM Canellos JP Peterson BA Randomized comparison of ABVD and MOPP/ABV hybrid for the treatment of advanced Hodgkin's disease: report of an Intergroup trial J Clin Oncol 2003 21 607 614 10.1200/JCO.2003.12.086 12586796

[B2] Evens AM Hutchings M Diehl V Treatment of Hodgkin lymphoma: the past, present, and future Nat Clin Pract Oncol 2008 5 543 556 10.1038/ncponc1186 18679394

[B3] Raemaekers JMM van der Maazen RWM Hodgkin's lymphoma: news from an old disease Neth J Med 2008 66 457 466 19075311

[B4] Kuderer NM Dale DC Crawford J Cosler LE Lyman GH Mortality, morbidity, and cost associated with febrile neutropenia in adult cancer patients Cancer 2006 106 2258 2266 10.1002/cncr.21847 16575919

[B5] Aapro MS Cameron DA Pettengell R Bohlius J Crawford J Ellis M Kearney N Lyman GH Tjan-Heijnen VC Walewski J Weber DC Zielinski C European Organisation for Research and Treatment of Cancer (EORTC) Granulocyte Colony-Stimulating Factor (G-CSF) Guidelines Working Party EORTC guidelines for the use of granulocyte-colony stimulating factor to reduce the incidence of chemotherapy-induced febrile neutropenia in adult patients with lymphomas and solid tumours Eur J Cancer 2006 42 2433 2453 10.1016/j.ejca.2006.05.002 16750358

[B6] Crawford J Dale JC Lyman GH Chemotherapy-induced neutropenia: risks, consequences, and new directions for its management Cancer 2004 100 228 237 10.1002/cncr.11882 14716755

[B7] Chirivella I Bermejo B Insa A Pérez-Fidalgo A Magro A Rosello S García-Garre E Martín P Bosch A Lluch A Optimal delivery of anthracycline-based chemotherapy in the adjuvant setting improves outcome of breast cancer patients Breast Cancer Res Treat 2009 114 479 484 10.1007/s10549-008-0018-1 18463977

[B8] Bosly A Bron D van Hoof A de Bock R Berneman Z Ferrant A Kaufman L Dauwe M Verhoef G Achievement of optimal average relative dose intensity and correlation with survival in diffuse large B-cell lymphoma patients treated with CHOP Ann Haematol 2008 87 277 283 10.1007/s00277-007-0399-y 17952688

[B9] Bonadonna G Valagussa P Moliterni A Zambetti M Brambilla C Adjuvant cyclophosphamide, methotrexate, and fluorouracil in node-positive breast cancer: the results of 20 years of follow-up N Engl J Med 1995 332 901 906 10.1056/NEJM199504063321401 7877646

[B10] Kwak LW Halpern J Olshen RA Horning SJ Prognostic significance of actual dose intensity in diffuse large-cell lymphoma: results of a tree-structured survival analysis J Clin Oncol 1990 8 963 977 2348230 10.1200/JCO.1990.8.6.963

[B11] Lyman GH Impact of chemotherapy dose intensity on cancer patient outcomes J Natl Compr Canc Netw 2009 7 99 108 19176210 10.6004/jnccn.2009.0009

[B12] Chand VK Link BK Ritchie JM Shannon M Wooldridge JE Neutropenia and febrile neutropenia in patients with Hodgkin's lymphoma treated with doxorubicin (Adriamycin), bleomycin, vinblastine and dacarbazine (ABVD) chemotherapy Leuk Lymphoma 2006 47 657 663 10.1080/10428190500353430 16690524

[B13] Crawford J Dale DC Kuderer NM Culakova E Poniewierski MS Wolff D Lyman GH Risk and timing of neutropenic events in adult cancer patients receiving chemotherapy: the results of a prospective nationwide study of oncology practice J Natl Compr Canc Netw 2008 6 109 118 18319047 10.6004/jnccn.2008.0012

[B14] Pettengell R Schwenkglenks M Leonard R Bosly A Paridaens R Constenla M Szucs T Jackisch C Impact of Neutropenia in Chemotherapy - European Study Group (INC-EU) Neutropenia occurrence and predictors of reduced chemotherapy delivery: results from the INC-EU prospective observational European neutropenia study Support Care Cancer 2008 16 1299 1309 10.1007/s00520-008-0430-4 18351398

[B15] Mosteller RD Simplified calculation of body-surface area N Eng J Med 1987 317 1098 10.1056/NEJM198710223171717 3657876

[B16] Cancer Therapy Evaluation Program Common Terminology Criteria for Adverse Events (CTCAE) Version 3.0 2006 http://ctep.cancer.gov/protocolDevelopment/electronic_applications/ctc.htm#ctc_40 accessed 20 July 2010

[B17] Dale DC McCarter GC Crawford J Lyman GH Myelotoxicity and dose intensity of chemotherapy: reporting practices from randomized clinical trials J Natl Compr Canc Netw 2003 1 440 454 19761076 10.6004/jnccn.2003.0038

[B18] Evens AM Cilley J Ortiz T Gounder M Hou N Rademaker A Miyata S Catsaros K Augustyniak C Bennett CL Tallman MS Variakojis D Winter JN Gordon LI G-CSF is not necessary to maintain over 99% dose-intensity with ABVD in the treatment of Hodgkin lymphoma: low toxicity and excellent outcomes in a 10-year analysis Br J Haematol 2007 137 545 552 10.1111/j.1365-2141.2007.06598.x 17459049

[B19] Boleti E Mead GM ABVD for Hodgkin's lymphoma: full-dose chemotherapy without dose reductions or growth factors Ann Oncol 2007 18 376 380 10.1093/annonc/mdl397 17071938

[B20] Chand VK Link BK Ritchie JM Shannon M Wooldridge JE Neutropenia and febrile neutropenia in patients with Hodgkin's lymphoma treated with doxorubicin (Adriamycin), bleomycin, vinblastine and dacarbazine (ABVD) chemotherapy Leuk Lymphoma 2006 47 657 663 10.1080/10428190500353430 16690524

